# Supplementary material for: Analysis of the polycystin complex (PCC) in human urinary exosome–like vesicles (ELVs)
Source: Sci Rep. 2020 Jan 30;10:1500. doi: 10.1038/s41598-020-58087-3 (PMC6992733; doi:10.1038/s41598-020-58087-3)
Supplement: Supplementary file 1 — Supplementary figures for Analysis of the polycystin complex. [file 41598_2020_58087_MOESM1_ESM.pdf]

Analysis of the polycystin complex (PCC) in human urinary exosome–like vesicles (ELVs).

Wendy A. Lea[1,+], Kerri McGreal[1,+], Madhulika Sharma[1,+], Stephen C. Parnell[1,2], Lesya Zelenchuk[1], M. Cristine Charlesworth[3], Benjamin J. Madden[3], Kenneth L. Johnson[3], Daniel J. McCormick[3], Marie C. Hogan[4], and Christopher J. Ward[1,*]

[1]The Jared Grantham Kidney Institute, University of Kansas Medical Center, Kansas City, KS 66160, USA.
[2]Department of Biochemistry and Molecular Biology, University of Kansas Medical Center, Kansas City, KS 66160, USA.
[3]Mayo Proteomic Core, Medical Sciences Building, Ms 3–121, Mayo Clinic, 200 First Street, SW Rochester, MN 55905, USA.
[4]Division of Nephrology, Department of Internal Medicine, Mayo Clinic, Rochester, USA.
[*]cward6@kumc.edu
[+]These authors contributed equally to this work

ABSTRACT

The polycystin–1 (PC1), polycystin–2 (PC2) and fibrocystin proteins, the respective products of the *PKD1*, *PKD2* and *PKHD1* genes, are abundant in urinary exosome–like vesicles (ELVs) where they form the polycystin complex (PCC). ELVs are $100\,\mathrm{nm}$ diameter membrane vesicles shed into the urine by the cells lining the nephron. Using MS/MS analysis of ELVs from individuals with *PKD1* mutations and controls, we show that in addition to the well-described GPS/GAIN cleavage event in PC1 at 3048 aa and the proprotein convertase cleavage (PPC) event in fibrocystin at 3616 aa, there are multiple other cleavage events in these proteins. The C–terminal 11 transmembrane portion of PC1 undergoes three cleavage events *in vivo*. The absence of peptides from the C–terminal cytoplasmic tail of fibrocystin implies a cleavage event close to its single TM domain prior to loading onto the ELVs. There is also evidence that the C–terminal tail of PC2 is also cleaved in ELVs . Native gel analysis of the PCC shows that the entire complex is $> 2\,\mathrm{MDa}$ in size and that N–terminal GPS/GAIN cleaved PC1 and PPC cleaved fibrocystin ectodomains can be released under non-reducing conditions and resolve at $300\,\mathrm{kDa}$. This paper shows that the three major human cystogene proteins are detectable in human urinary ELVs and that all three undergo post-translational proteolytic processing. Human urinary ELVs may be a useful source of material in the search for proteins that interact with the PCC.

Introduction

Autosomal dominant polycystic kidney disease (ADPKD) has a prevalence of between 1:400 and 1:1000 individuals[1]. The most likely prevalence is 1:800, implying that there are 390,000 individuals living with ADPKD in the USA of which 30,000 require renal replacement therapy, at a yearly cost of $80,000 per patient and a minimum economic burden of $2.4 billion. Among affected individuals, 85% of the mutations found by conventional Sanger sequencing are in the *PKD1* gene and 15% in the *PKD2* gene. About 9–10% of individuals with clinical ADPKD have no mutation detected (NMD) for either gene. Some of these may have changes in the Glucosidase IIα subunit (GANAB)[2] or in yet to be identified *PKD* gene(s)[3] [pkdb.mayo.edu]. Mutations in *PKD1* and *PKD2* have a similar clinical phenotype characterized by the slow development of multiple fluid-filled kidney cysts, leading to end stage renal failure at an average age of 54yrs in *PKD1* and 74yrs in *PKD2*[4]. The products of these genes, polycystin–1 (PC1) [P98161 PKD1_HUMAN], polycystin–2 (PC2) [Q13563 PKD2_HUMAN] and fibrocystin [P08F94 PKHD1_HUMAN] are large membrane spanning proteins with lengths of 4302, 968 and 4074 aa, respectively (see Figure 1a for the overall domain structure). These proteins have all been shown to be present in the urine in 100 nm diameter membrane-bound vesicles, i.e. exosome–like vesicles (ELVs)[5]. Yeast two hybrid screening together with directed over-expression work in cell lines determined that PC2 could interact with both PC1 and fibrocystin[6–8]. Furthermore, mouse breeding experiments showed that heterozygous *Pkd1* alleles could exacerbate cystic disease on either a homozygous *Pkhd1* or heterozygous *Pkd2* background[9–11]. Thus, the biochemical and genetic data strongly suggests that these molecules are involved in a higher order arrangement, the polycystin complex (PCC)[10,12].
Our initial work using quantitative proteomics in ELVs derived from individuals with *PKD1* mutations and controls indicated that PC1 and PC2 were decreased in individuals with a mutation[5]. Fibrocystin did not reach significance using the stringent

statistical techniques that were applied but did show a decreasing trend in q–value (q=0.063 and a *PKD1*/normal ratio of 0.67). Cell surface hyaluronidase (CEMIP2_TMEM2 [Q9UHN6 CEIP2_HUMAN]), a fibrocystin homologue, was increased in individuals with *PKD1* mutations[13]. These observations suggested that a decrease in PC1, secondary to germline mutation, reduced the amount of the mature PCC and its components. In this scenario, PC1 was a 'scaffold' molecule around which a higher order multi-component complex of PC2, fibrocystin and other interactors were assembled. This idea was compatible with a genetic analysis showing that *Pkd1* (PC1) dosage was the main determinant of cystogenesis where low amounts of PC1 could not be complemented by high *Pkd2* (PC2) or *Pkhd1* (fibrocystin) levels[14]. The doubling of CEMIP2/TMEM2 abundance in ELVs led us to hypothesize that this fibrocystin homologue might be a regulated by the PCC.
When both PC1 and fibrocystin were overexpressed in cell culture systems both proteins underwent a series of proteolytic modifications. PC1 was cleaved in the GPS/GAIN domain at the **HL↑T** autocleavage site (aa 3048) and generated a large N–terminal extracellular ectodomain and an 11 TM spanning C–terminal section[15]. The published literature proposes that the C–terminal portion of PC1 undergoes two further cleavage events: the first between TM V–VI generated a 100 kDa C–terminal fragment which contained the last 6 TM domains (TM VI–XI), and a second event in the cytoplasmic tail released a non membrane bound 14 kDa fragment that could translocate to the nucleus[16,17]. The literature also suggests that there was another cleavage event which released a 34 kDa C–terminal fragment[18]. In the case of fibrocystin, *in vitro* work delineated a pro-protein convertase site (PPC) at 3616 aa that generated a large N–terminal ectodomain. A further cleavage event C–terminal to the TM domain released a the cytoplasmic tail, which like the C–terminus of PC1, translocated to the nucleus[19,20]. All of these observations were dependent on the over-expression of the subunits of the PCC usually without their cognate partners (some of which may be unknown). Here we probe the native PCC in ELVs and show that some of the above cleavage events do occur in the native complex, but others do not. There are also novel proteolytic events as well. These have profound implications for the higher order structure of the PC1/PC2 complex.

Results

The following data refer to the polycystin complex (PCC) in human urinary ELVs. We revisited tryptic peptide data from a study comparing ELVs from 13 individuals with *PKD1* mutations and 18 individuals with normal kidneys[5]. In this study, urinary ELVs prepared by ultracentrifugation on a 5–30% sucrose D_2O gradient were resolved on a 4–12% SDS PAGE with each individual run in a separate lane. Each lane was then analyzed by sectioning into 10 slices A–J according to molecular mass, with A being the highest and J the lowest. In our new analysis of this data, we compared levels of PC1, PC2, fibrocystin and CEMIP2/TMEM2 peptides in individuals with and without *PKD1* mutations and showed that PC1, PC2 and fibrocystin peptides were decreased along their entire length in individuals with *PKD1* mutations, whereas CEMIP2/TMEM2 peptides were increased along their entire length of the polypeptide, Figure_1 and Supplemental_Figure_1 (Data in Supplemental_Database_1) . The peptide data shows that the extracellular N–termini of PC1 and fibrocystin are mainly seen in gel section A, 270 kDa–500 kDa. After amino acid 3048 in PC1, the site of GPS/GAIN cleavage, peptide intensity appears to decrease, and peptides are found at lower molecular weights spread over a number of gel slices. In the case of fibrocystin, the N–terminal portion of the molecule down to the PPC site at 3616 aa appears mainly in gel section A. Crossing the PPC, peptides appear in gel sections E and F between 40 kDa–70 kDa, verifying cleavage. PC2 appears to run as a smear across gel slices A, B and C, 90 kDa–500 kDa range. CEMIPS_TMEM2 shows no sign of a cleavage event, and all peptides are in gel slice B 140 kDa–270 kDa.

Cleavage of the C–terminal 11 transmembrane spanning portion of PC1

Most tryptic peptides ending at an amino acid less than 3048 aa (the GPS/GAIN cleavage site) were in gel slice A (the ectodomain) whereas peptides after this point were spread over sections (B, C, D and E), implying that the C–terminal 11 TM domains of PC1 existed in more than one proteolytically cleaved form, Supplemental_Database_1. The MS/MS peptide intensity data showed that there was an excess of N–terminal ectodomain PC1 versus the C–terminal TM region. Peptides in slice A that had a stop position < 3048 aa had an intensity 1.4 fold that of the average intensity seen in other slices (B–J) with a stop position > 3048 (here peptides intensities present in multiple slices were summed and the average for all peptides determined). This suggested that ectodomain PC1 uses another mechanism besides interaction through the GPS/GAIN domain in order to remain bound to ELVs as the two fragments are not in a 1:1 stoichiometry. We expected that there would be a decrease in the amount of sheddable ectodomain versus membrane anchored C–terminal PC1 as the ELVs underwent three ultracentrifugation steps – but the opposite is true, Figure_1.
We were particularly interested in the putative cleavage events in the C–terminus portion of PC1. We used a rat monoclonal antibody (MC-Ab) E8 raised to mouse PC1 (3682..3882 aa) between TM VI–VII, and a mouse MC-Ab PKS–A (161F IgG1κ), which was epitope mapped to the C–terminal tail of PC1 between TM11 and coiled-coil domain (4071..4156 aa) to probe the proteolytic events in the C–terminus, Figure_2. Both E8 and PKS–A(161F) recognize a large 157 kDa (A^{glyc}) band which is reduced to 145 kDa ($A^{n\text{-}glyc}$) by PNGase F, but not Endo H, implying post-Golgi maturation of the protein (^{glyc} refers

to the glycosylated form and $^{n\text{-}glyc}$ not glycosylated). The $A^{n\text{-}glyc}$ form of PC1 was responsible for about 40% of the total signal. Both antibodies also detected a 96 kDa band (B^{glyc}) and an 80 kDa band (C^{glyc}) which respectively reduced to 81 kDa ($B^{n\text{-}glyc}$) and 70 kDa bands ($C^{n\text{-}glyc}$) in the PNGase F lane. The 11 kDa difference between $B^{n\text{-}glyc}$ and $C^{n\text{-}glyc}$ was due to a cleavage event in the C–terminal tail, which was predicted to remove the coiled-coil region. At lower molecular masses, the two antibodies recognize different fragments: PKS–A (161F) was cognate to a 62 kDa band (D^{glyc}) in the glycosylated lane and a 52 kDa band ($D^{n\text{-}glyc}$) in the PNGase F lane. The $D^{n\text{-}glyc}$ fragment underwent the C–terminal trimming of 11 kDa as there was a 41 kDa ($E^{n\text{-}glyc}$) fragment visible on the gel, especially under a long exposure on a 4–12% MOPS gel, Figure_2b. E8 recognized a 31 kDa (F^{glyc}) band in the glycosylated lanes and a 29 kDa band ($F^{n\text{-}glyc}$) in both Endo H and PNGase F lanes. There are three NX[STC] glycosylation sites in the C–terminal GPS cleaved portion of PC1, and the cluster in the region between TM–VI and VII in the TOP domain (3738, 3790 and 3845 aa). We think that the 31 kDa (F^{glyc}) E8 fragment has a single Endo H sensitive site with 2 kDa of carbohydrate at 3738 aa, and the 62 kDa (D^{glyc}) PKS–A (161F) product has two mature N–linked carbohydrate side chains with a total mass of 10 kDa, at aa 3790 and 3845. Thus, there must be a cleavage site in the TM–VI, TM–VII loop between the first and second glycosylation sites, 3738..3790 aa. The 52 kDa $D^{n\text{-}glyc}$ band seen by PKS–A (161F) and the 29 kDa $F^{n\text{-}glyc}$ band seen by E8 add up to 81 kDa $B^{n\text{-}glyc}$ band seen by both, implying that they are contiguous. The 29 kDa $F^{n\text{-}glyc}$ fragment must extend N–terminally to the TM–IV/TM–V hairpin, implying that monoclonal E8 is cognate to the first half of the TM–VI, TM–VII loop in the TOP domain. Altogether, these data suggest that in the native complex, over 50% of the C–terminal PC1 molecules undergo three distinct proteolytic events. Furthermore, PC1 has one Endo H sensitive sugar moiety on the first N–linked carbohydrate site and two mature complex carbohydrates on the next two N–linked carbohydrate sites.

Mass spectrometry of the C–terminal 11–TM spanning region of PC1

In order to verify the antibody mapping work with E8 and PKS–A (161F), we deglycosylated ELVs with PNGase F and ran both MES and MOPS gels recovering gel slices A^{ms}–E^{ms}, (which map to $A^{n\text{-}glyc}$-$F^{n\text{-}glyc}$, excluding $E^{n\text{-}glyc}$ which is dropped so that E^{ms} maps to $F^{n\text{-}glyc}$) for trypsin proteolysis and LC–MS/MS analysis, Figure_3. As expected few peptides were recovered for the ectodomain of PC1 up to aa 3048. Gel slice A^{ms} ($A^{n\text{-}glyc}$) at 145 kDa has peptides spanning the entire 11–TM spanning region including a peptide generated by tryptic digestion together with GPS/GAIN cleavage **TAFGASLFVPPSHVR**. This formally proves that there is a naturally occurring cleavage event at the **HL↑T** autocleavage site at aa 3048, Figure_3c[15]. Slices B^{ms} ($B^{n\text{-}glyc}$) and C^{ms} ($C^{n\text{-}glyc}$) contain peptides from the entire C–terminal region yet run at 81 kDa and 70 kDa implying that a fragment extending from the GPS cleavage site to TM IV–V is also present at these masses, but not detected with the antibodies used (there are no antibodies available for this segment).

Slice D^{ms} ($D^{n\text{-}glyc}$) has peptides from the last 5 TMs again as predicted from the antibody data (blue box, Figure_3b) whereas slices E^{ms} ($F^{n\text{-}glyc}$), Control slice (Ctrl) and 11 kDa have no detectable peptides, Figure_3. This implies that the 11 kDa trimming event occurs before loading of the PCC into the exosome. (Data in Supplemental_Database_1A).

Analysis of PC2 shows that it has mature glycosylation on ELVs and that it undergoes C–terminal trimming

PC2 ran as a smear in the range 160 kDa–260 kDa which was PNGase F but not Endo H sensitive, showing that it has undergone post-Golgi carbohydrate modification. This was an unusual finding as this protein has until now been reported to be exclusively Endo H glycosylated[21,22]. The smearing may be due to the hydrophobic nature of PC2 as we did not detect any PC2 peptides with the hall marks of ubiquitylation, though we did observe ubiquitylation of polyubiquitin-B, smoothened, filamin and VPS4A[23]. The smearing of PC2 was independent of the temperature (20–90°C) used to treat the sample prior to loading. Furthermore, the pattern observed in ELVs was different to that seen in human renal cortical tubular epithelial (RCTE) cells which had a strong definite band at 260 kDa representing the dimeric form of PC2, and weaker 120 kDa and 500 kDa bands representing monomeric and tetrameric PC2, respectively[22]. We believe that ELV PC2 is present in a cholesterol rich domain within the ELV and interacts strongly with lipids giving rise to the smearing which is not seen in PC2 from cells, Supplemental_Figure_2.

There were four C–terminal peptides in gel slice I which map to the last 17.8 kDa of the PC2 molecule implying that there was a cleavage event in the C–terminal tail which must occur N–terminal to the coiled-coil domain (833..872 aa). This is because the most N–terminal peptide in these fractions was **SLDDSEEDDDEDSGHSSR**, 807..825 aa, but was of poor quality. The next peptide of high quality was **GSISSGVSYEEFQVLVR**, 828..844 aa, present in sections A–D and I–J. The peptides in I–J have an average intensity 13% of those in sections A–D, Supplemental_Figure_3. The implication is that a minority of PC1 and PC2 undergo proteolytic processing that removes their respective coiled-coil domains, which mediate part of the PC1/PC2 interaction[6].

Analysis of fibrocystin confirms two cleavage events

Our analysis of fibrocystin, the product of the *PKHD1* gene, shows that most peptides with stop positions less than 3616 aa (the PPC site) were present in the highest band, band A. The average intensity of peptides decreased after the PPC site so that the intensity of peptides running in bands B–J with a stop position after 3616 aa was 58% of the average value for peptides with a stop position less than 3616 aa in band A. Thus, there is a deficit of fibrocystin spanning the region from the PPC site to the TM, and this raises questions as to how ectodomain fibrocystin remains bound to ELVs after three ultracentrifugation runs. Furthermore, there were very few peptides after the beginning of the single TM domain, at 3859 aa. There was a single C–terminal tail peptide in three different gel slices (D,E and F) with a mean intensity of 5.1% of the average intensity of peptide seen in section A (**KEDTVVGEDMR**, 3921..3932). There were 16 potentially detectable tryptic peptides in the C–terminal tail of fibrocystin that are within range of our MS/MS analysis, (Data in Supplemental_Database_2). In short, there was a deficit of peptides after the PPC site and basically no peptides after the TM domain. These data suggest that fibrocystin underwent C–terminal tail cleavage prior to loading into ELVs.

Analysis of the ELV PCC

Western analysis of the extracellular portions of PC1 (450 kDa glycosylated), fibrocystin (500 kDa glycosylated), PC2(160 kDa–260 kDa) and CEMIPS/TMEM2 (200 kDa glycosylated) molecules showed that all had mature Endo H-resistant glycosylation, Figure_4. Native gel analysis with a mild detergent, lauryl maltoside neopentyl glycol (LMNG), allowed the high molecular weight (2 MDa) PCC to migrate a short distance into the gel. Small amounts of ectodomain PC1 and fibrocystin were released under these non-reducing conditions and migrated at about 300 kDa. These ectodomains migrate faster in the native gels than the SDS PAGE gels as they still have their tertiary structure and less fluid drag than the denatured unwound proteins.

We attempted to solubilize this high molecular weight material (2 MDa) with a range of different alkyl glycoside detergents in an attempt to dissociate the membrane bound complex into a number of sub-complexes, but with little success. However, we found that low amounts of SDS in the range of 0.2–0.8% (without heating) released most of the N–terminal PC1, fibrocystin and the CEMIPS_TMEM2 molecules. However, PC2 and the C-terminal 11 TMs of PC1 remained tightly associated with a very large complex $>$ 2MDa, implying that they interact strongly with the cholesterol-rich membrane of the exosome, Figure_4b and c. We used this range of SDS as $<$ 0.2% SDS did not release PC1 ectodomain whereas levels $>$ 0.8% SDS dissociated the entire complex into individual subunits[24].

We focused on the N–terminal PC1 and fibrocystin molecules and applied MS/MS analysis to 16 sequential gel slices targeting the region in which they resolve (in this case, ELVs were resolved in the presence of 0.1% lauryl maltoside neopentyl glycol (LMNG), 0.1% taurocholic acid and 0.1% amphipol). These data showed that N–terminal PC1 and fibrocystin ran in different gel slices (10 and 8, respectively, fibrocystin ran at a higher Mwt than PC1) and that there was therefore no evidence of a direct physical interaction between the two molecules under non denaturing gel electrophoresis. Furthermore, the MS/MS data and the detergent data strongly suggest that the ectodomains of PC1 and fibrocystin were non-covalently attached to the membrane of the ELV with an interaction robust enough to withstand multiple washing and ultracentrifugation runs, Supplemental_Database_2.

Discussion

The PCC is a large complex composed of at least three proteins found in ELVs , PC1, PC2 and fibrocystin. The PCC is also present on the primary cilium and perhaps elsewhere in the cell and may not have exactly the same partners and processing steps as the PCC in the ELV[25]. This paper focuses on the PCC in ELVs at it is a rich source of the proteins and has low complexity when compared with cells or tissue.

All PCC proteins have mature post-Golgi glycosylation and exist in a large complex that resolves at a molecular mass of $>$ 2 MDa on non denaturing acrylamide gel (blue native gel). It is possible to release the ectodomains of fibrocystin and PC1 using mild detergent, but PC2 and C–terminal PC1 remain in a very high molecular weight complex which was resistant to all but the most denaturing detergents (SDS).

Revisiting the work of Hogan *et al* which used denaturing SDS PAGE and pure, THP-free, ELVs derived from 18 normal and 13 individuals with defined *PKD1* mutations, we showed that at the peptide level, PC1, PC2 and fibrocystin were all decreased in those with a mutation[5]. These data strongly suggest that at the exosome level, there was a deficit of the PCC in the ADPKD state. We think that the PCC was dependent on PC1 for assembly and that the reduction of this 'scaffold' protein was directly responsible for the reduction of PC2 and fibrocystin in the ELVs. This idea fits with the analysis of the genetic interaction between *Pkd1*, *Pkd2* and *Pkhd1* which showed that *Pkd1* dosage was the most important contributor to cystogenesis and that it cannot be complemented by over-expression of *Pkd2* or *Pkhd1*[14]. The increase in CEMIPS_TMEM2 was more difficult to explain. One idea was that the mature PCC interacts with CEMIPS_TMEM2 and modulates its loading onto ELVs . A reduction of PCC would allow an increase in CEMIPS_TMEM2. CEMIPS_TMEM2 is an active neutral pH hyaluronidase, so the PCC may have a role in controlling the amount of this enzymatic activity on ELVs[13].

We confirmed the work of Qian *et al* who showed that PC1 undergoes an autoproteolysis step in the GAIN/GPS region at 3048 aa (**HL↑T**) by detecting the GPS cleavage product **TAFGASLFVPPSHVR**, Figure_3 c and d[15]. This generates two large products in PC1: the 323 kDa unglycosylated (450 kDa glycosylated) N–terminal fragment (ectodomain) and a 157 kDa glycosylated A^{glyc} (145 kDa unglycosylated $A^{n\text{-}glyc}$) C–terminal fragment. In ELVs, about 60% of the C–terminal fragment undergoes three proteolytic events that have not been observed in over-expressed PC1. Using the N–linked glycosylation sites as milestones, we were able to map two cleavage events: the first close to the TM–IV and TM–V hairpin, a tight turn between the two transmembrane helices with two turn–like amino acids (**PP**), and the second between the first two glycosylation sites in the large polycystin loop between TM–VI and TM–VII (3738..3790 aa), the TOP domain. We further predicted that there is a 11 kDa trimming event at the C–terminus of the protein that would remove the C–terminal coiled-coil region.
Using directed LC–MS/MS on the gel slices containing the C–terminal cleavage products, we were able to confirm that product $A^{n\text{-}glyc}$ contained the entire C-terminal portion of PC1. Products $B^{n\text{-}glyc}$ and $C^{n\text{-}glyc}$ run at approximately half the molecular weight of $A^{n\text{-}glyc}$ but contain the same complement of peptides as $A^{n\text{-}glyc}$ again suggesting that the C–terminus is cut in two equal portions at about TM–IV/TM–V hairpin. There is an N–terminal fragment of about 70 kDa extending from the GPS/GAIN cleavage to TM–IV/TM–V running together with the C–terminal fragment that is visualized with MCs E8 and PKS–A (161F) which extends from TM–V to the coiled coil. $D^{n\text{-}glyc}$ has the peptide range predicted by the antibody work, from about the middle of the TM–VI/TM–VII 'polycystin loop' (TOP domain) to the extreme C–terminus of the protein. We attempted to find peptides for E^{ms} ($F^{n\text{-}glyc}$) but failed to find peptides in the range TM–V to the middle of the TM–VI/TM–VII loop. This may be because $F^{n\text{-}glyc}$ is not abundant, perhaps being a transient product, and can only be observed with the excellent E8 antibody. We were unable to detect PC1 peptides in the range 11 kDa implying that the C–terminal cleavage event in $C^{n\text{-}glyc}$ might happen prior to the packaging of the PCC into the ELVs . This would certainly support the idea that the coiled coil region is cleaved and translocated to the nucleus[16]. We were able to detect peptides C–terminal to the coiled coil domain in $A^{n\text{-}glyc}$ and $D^{n\text{-}glyc}$. In all, the MS/MS analysis supports the antibody mapping work.
These data suggested that the 100 kDa fragment seen with *in vitro* over-expression systems might be similar to our 96 kDa glycosylated, B^{glyc}, (81 kDa unglycosylated protein $B^{n\text{-}glyc}$) though we suggest a cleavage event in the TM–IV/TM–V hairpin and not between TM–V and TM–VI as suggested by these authors[17]. Our 11 kDa trimming event may be similar to the 14 kDa cleavage seen by Low *et al*[16]. Interestingly, the 81 kDa B and 70 kDa C forms of PC1 would be predicted to span the membrane seven times analogous to a classical seven TM spanning molecule. This fits with the idea that PC1 is a G–protein coupled receptor as well as an ion channel subunit[26,27].
We detected PC2 with mature post-Golgi N–linked glycosylation but the channel runs as a smear pre and post deglycosylation. ELV PC2 runs as a smear at 160 kDa–260 kDa, from the size of the 260 kDa dimer downward. In human RCTE cells PC2 resolves as tetramer 500 kDa, dimer 260 kDa and monomer 120 kDa, implying that there are different forms of the protein in exosomes versus cells, Supplemental_Figure_2. We think that ELV PC2 runs as a smear as it is interacting with lipids (possibly cholesterol) from the ELV membrane. One possibility is that the ELV form of PC2 is due to alternative splicing of the *PKD2* gene (for example, the *PKD2Δ7* form of the PC2 cDNA described by Hackmann *et al*[28]). This possibility is difficult to probe with the current data set as the spliced region (exons 6–8) encode hydrophobic residues lacking lysine (K) and arginine (R) residues that are appropriately positioned to generate tryptic peptides in the mass range for the mass spectrometer. Future work would use enzymes such as chymotrypsin to probe these regions in detail. In summary, the smearing of ELV PC2 is probably secondary to its hydrophobic nature as the MS/MS failed to detect evidence of ubiquitinylation (lysine modified by diglycine). We also detected C–terminal peptides in gel sections I and J which were $<$ 24 kDa, implying a cleavage event between the EF–hand 750..785 aa and the coiled-coil domain at 833..872 aa. These represent about 10% of the protein.
Overall, these data have implications for the structure of the PC1/PC2 complex which was recently generated by over-expressing the region containing the terminal 11 TM domains of PC1 ($PKD1^{3049..4169}$) and the six TM domains of PC2 ($PKD2^{185..723}$). In this study, a mammalian cell line (human embryonic kidney (HEK) 293F) was used to express both proteins and the higher order complex was purified using sequential epitope tags. PC1 was purified first using a FLAG tag and then the PC1/PC2 complex retrieved using a further Strept tag affinity step to generate the final complex[29]. The recombinant PC1/PC2 complex, on which the structure was determined, has not undergone internal cleavage events implying that the presented framework structure may require modification in the light of our findings.
Fibrocystin appears to be cleaved at the PPC site at 3616 aa and undergoes a cleavage event at the TM domain[19,20]. The region between the PPC site and the TM appears to be underrepresented (58% of the level of the extracellular portion $<$ 3616 aa), and the cytoplasmic C–terminal tail was at even lower levels and was probably not present at all (<5.1%), implying that it was removed prior to packaging into the ELV. This was compatible with the finding that nephrogenesis and hepatogenesis are completely normal in mice lacking the C–terminal cytoplasmic domain, the $Pkhd1^{\Delta 67}$ mice[30]. The evolutionarily close fibrocystin–L protein (*PKHD1L1* Q86WI1 (PKHL1_HUMAN)) has only nine C–terminal tail amino acids suggesting that some members of the fibrocystin family can function without the cytoplasmic portion of the protein[31]. Furthermore, there is data suggesting that the C–terminal tail of fibrocystin is cleaved and translocates to the nucleus[20].

The cleaved ectodomains of both PC1 and fibrocystin appear to be over-represented versus the quantity of TM spanning regions from each protein. We suggest that for both proteins, there must be a mechanism that is independent of interaction with their TM domains that allows an excess of the ectodomain to remain bound to the exosome after the ultracentrifugation steps. One idea is that there may be an amphipathic α–helix in the ectodomain of PC1 which allows the N-terminus to remain associated with the ELV membrane similar to that seen in EMR2/ADGRE2, a protein with a GPS/GAIN domain[32]. We were unable to detect such an in-plane membrane anchor using AmphipaSeeK in PC1[33].
CEMIPS_TMEM2, a fibrocystin homologue and extracellular hyaluronidase increased in individuals with *PKD1* mutations, appears not to undergo proteolytic processing. It has 20 kDa of mature N–linked glycosylation and was present in a very large >2MDa complex indistinguishable from the PC1/PC2 complex on native gels. However, it was easily released from the PCC using mild SDS treatment suggesting that CEMIPS_TMEM2 may be peripheral to the PCC, and binding occurs via a low affinity and perhaps transient interaction, Figure 4.
The PCC itself appears to be a very large complex that would not resolve on 4% acrylamide gels. This may be because the PCC is embedded in the cholesterol-rich exosome and that the conditions necessary to solubilize it are close to those that will cause the multi-protein complex to dissociate[24,34]. However, both the N–terminal ectodomains of fibrocystin and PC1 could be released from the complex by gentle SDS treatment or with a cocktail of mild detergents, i.e. 1% taurocholic acid and 0.5% lauryl maltoside neopentyl glycol, Figure 4b.
These data suggest that urinary ELVs are a rich source of the PCC and that they can be used to delineate the post-translational modifications the members of the complex undergo. Urinary ELVs might also be useful in the search for new members of the PCC and when confirmed these would immediately become candidates for the missing PKD genes.

Methods

Database

The entire data set is available at the ProteomeXchange.
ftp://ftp.pride.ebi.ac.uk/pride/data/archive/2014/12/PXD001075 accession PXD001075.

Antibodies

Antibodies used were anti–N–terminal PC1 7e12 (IgG1κ anti–LRR)[35], anti–C–terminal PC1 PKS–A (161F, IgG1κ)) epitope mapped between amino acids (4070..4199)[36]. PC2 antibodies (H-280): sc-25749 rabbit polyclonal to 689..968 aa (Santa Cruz), YCE2 Polycystin-2 Antibody (YCE2): sc-47734 (IgG2aκ)[37]. Fibrocystin antibody Ab 18 (IgG2aκ) to the extracellular portion of the molecule and epitope mapped between residues 143..238 aa[38]. Anti–CEMIPS_TMEM2 polyclonal antibody (HPA044889 Sigma) produced in rabbit to the cytoplasmic region of CEMIPS_TMEM2 1..69 aa. The E8 antibody, rat monoclonal polycystin-1 (E8) Antibody (E8A-8C3C10 and E8B-8C3D11) was provided by the NIH NIDDK sponsored Baltimore Polycystic Kidney Disease Research and Clinical Core Center, P30DK090868 fqian@medicine.umaryland.edu http://www.baltimorepkdcenter.org/.

Western analysis

Membrane protein samples were resolved on NuPAGE 4–12% Bis-Tris or 3–8% Tris-acetate gels (ThermoFisher Scientific) via SDS-PAGE (XCell SureLock Mini-cell, Thermofisher Scientific). Proteins were transferred onto a 0.45 µm pore size PVDF membrane (Millipore), after wetting in 95% ethanol, using 10 mM CAPS pH 11.0 buffer with 0.01% SDS, 1 hour at 70 V. After blocking the membrane with 5% milk TBS 0.1% Tween–20, the corresponding primary antibody was applied at 1:2000 in 'half–block' 2.5% milk TBS, 0.1% Tween–20 from 1 hour 20°C to over night 4°C. Blots were washed 3x 5 minutes each then probed with the appropriate HRP–labeled secondary from Southern Biotech (https://www.southernbiotech.com) at 1:2000 in 'half-block'. For example, 7e12 is a mouse IgG1κ monoclonal antibody and would be probed with 1070-05 Goat Anti–Mouse IgG1, Human ads-HRP. After washing 3 times in TBS 0.1% Tween–20, blots were subsequently imaged on an AI600 imager (GE Healthcare) using HyGLO quick spray as the detection reagent (Denville Scientific Inc.).

Equipment and settings

Western data was acquired using a 16 bit CCD camera AI600 imager (GE Healthcare). Images showed the entire length of the gel and were cropped using GNU Image Manipulation Program (GIMP) https://www.gimp.org/. Figures were assembled in Inkscape https://inkscape.org/. Contrasting and enhancement restricted to using the 'curves' function in GIMP alone. Exposure times are in the metadata of the uploaded supplemental .tif files.

Production of ELVs from urine

ELVs were purified using the method of Chen *et al*[5,38,39]. Briefly, 360 mL of urine was centrifuged at 4000g in a Sorvall Evolution RC centrifuge (SLA–1500 rotor) for 15 minutes and the cellular pellet was discarded. The supernatant was then

centrifuged at a RCF_{max} 136000g for 1hr (35000rpm) in a Sorvall T–647.5 (wX+ Ultra–centrifuge, Sorvall) and the pellet retained. The pellet contains exosomes and the abundant protein Tamm Horsfall Protein (THP). The pellet was resuspended in 0.5 mL of 0.25M sucrose 20 mM MES pH 6.0 Complete®–EDTA free proteinase inhibitor (Sigma 11873580001) and layered onto a 5–30% sucrose D_2O gradient and centrifuged at a RCF_{avg} 202000g (k–factor=137.3) for 24hrs, in a TH641 rotor in a wX+ Ultra–centrifuge Sorvall. Under these conditions the THP pellets and exosomes resolve in three distinct populations. We harvested the ELV fraction B which resolves a refractive index of η=1.3530. Fraction B ELVs were then concentrated in an Amicon Ultra–15 50 kDa (UFC905024) concentrator 10–20x and this resulted in 0.5 mL of pure ELVs from 360 mL of starting urine.

Gel slice proteomics

The protocol used for in slice trypsin digest and LC–MS/MS is described in Hogan *et al.*[5].

Native gel analysis

Due to the large size of the proteins under study we used 3–8% gradient or 4% slab polyacrylamide gels without stacking gels as these would cause 'hang up' of protein at the stacking/running gel interface. We followed the protocol of Wittig *et al*[40]. Cathode buffer was 50 mM Tricine, 7.5 mM Imidazole pH 7.0, anode buffer was 25 mM Imidazole pH 7.0, gel buffer was 25 mM Imidazole, 500 mM 6–aminohexanoic acid. The detergents used were quoted in the text, the charge conferring agent was Coomassie blue G–250 5 $mg\,mL^{-1}$ or Amphipol A8–35 (25 $mg\,mL^{-1}$) (Anatrace A835) or taurocholic acid 0.1%. Gels were run over night 18hrs at 17 V at 4 °C, in Novex 1.5 mm cassettes (NC2015) on Xcel SureLock® gel apparatus. Western blotting was performed as above except that the transfer period was extended — 10 mM CAPS pH 11.0 buffer with 0.01% SDS for 7 hours at 30 V at 4°C.

Statistics

Statistic were performed using R https://www.r-project.org/, using packages dplyr
http://dplyr.tidyverse.org, https://github.com/tidyverse/dplyr
and ggplot2 for graphics
http://ggplot2.tidyverse.org, https://github.com/tidyverse/ggplot2 AmphipaSeeK was utilized to search for amphipathic sequences in PC1 and fibrocystin
https://npsa-prabi.ibcp.fr/cgi-bin/npsa_automat.pl?page=/NPSA/npsa_amphipaseek.html

References

1. Iglesias, C. G. *et al.* Epidemiology of adult polycystic kidney disease, olmsted county, minnesota: 1935-1980. *Am J Kidney Dis* **2**, 630–639 (1983).
2. Porath, B. *et al.* Mutations in ganab, encoding the glucosidase iiα subunit, cause autosomal-dominant polycystic kidney and liver disease. *The Am. J. Hum. Genet.* **98**, 1193–1207 (2016).
3. Rossetti, S. *et al.* Comprehensive molecular diagnostics in autosomal dominant polycystic kidney disease. *J Am Soc Nephrol* **18**, 2143–2160 (2007).
4. Orskov, B., Sørensen, V. R., Feldt-Rasmussen, B. & Strandgaard, S. Improved prognosis in patients with autosomal dominant polycystic kidney disease in denmark. *Clin J Am Soc Nephrol* **5**, 2034–2039 (2010). DOI 10.2215/CJN.01460210.
5. Hogan, M. C. *et al.* Identification of biomarkers for pkd1 using urinary exosomes. *J. Am. Soc. Nephrol.* ASN–2014040354 (2014).
6. Qian, F. *et al.* PKD1 interacts with PKD2 through a probable coiled-coil domain. *Nat Genet.* **16**, 179–83 (1997).
7. Wang, S. *et al.* Fibrocystin/polyductin, found in the same protein complex with polycystin-2, regulates calcium responses in kidney epithelia. *Mol Cell Biol* **27**, 3241–52 (2007).
8. Kim, I. *et al.* Polycystin-2 expression is regulated by a pc2-binding domain in the intracellular portion of fibrocystin. *J Biol Chem* **283**, 31559–31566 (2008). DOI 10.1074/jbc.M805452200.
9. Olson, R. J. *et al.* Synergistic genetic interactions between pkhd1 and pkd1 result in an arpkd-like phenotype in murine models. *J. Am. Soc. Nephrol.* ASN–2019020150 (2019).
10. Garcia-Gonzalez, M. A. *et al.* Genetic interaction studies link autosomal dominant and recessive polycystic kidney disease in a common pathway. *Hum Mol Genet.* **16**, 1940–1950 (2007). DOI 10.1093/hmg/ddm141.

11. Wu, G. *et al.* Trans-heterozygous pkd1 and pkd2 mutations modify expression of polycystic kidney disease. *Hum. molecular genetics* **11**, 1845–1854 (2002).

12. Wu, G. *et al.* Trans-heterozygous pkd1 and pkd2 mutations modify expression of polycystic kidney disease. *Hum Mol Genet.* **11**, 1845–1854 (2002). (eng).

13. Yamamoto, H. *et al.* A mammalian homolog of the zebrafish transmembrane protein 2 (tmem2) is the long-sought-after cell surface hyaluronidase. *J. Biol. Chem.* jbc–M116 (2017).

14. Fedeles, S. V. *et al.* A genetic interaction network of five genes for human polycystic kidney and liver diseases defines polycystin-1 as the central determinant of cyst formation. *Nat. genetics* **43**, 639–647 (2011).

15. Qian, F. *et al.* Cleavage of polycystin-1 requires the receptor for egg jelly domain and is disrupted by human autosomal-dominant polycystic kidney disease 1-associated mutations. *Proc Natl Acad Sci U S A* **99**, 16981–6 (2002).

16. Low, S. H. *et al.* Polycystin-1, stat6, and p100 function in a pathway that transduces ciliary mechanosensation and is activated in polycystic kidney disease. *Dev. cell* **10**, 57–69 (2006).

17. Woodward, O. M. *et al.* Identification of a polycystin-1 cleavage product, p100, that regulates store operated ca2+ entry through interactions with stim1. *PloS one* **5**, e12305 (2010).

18. Chauvet, V. *et al.* Mechanical stimuli induce cleavage and nuclear translocation of the polycystin-1 c terminus. *J. Clin. Investig.* **114**, 1433 (2004).

19. Kaimori, J.-Y. *et al.* Polyductin undergoes notch-like processing and regulated release from primary cilia. *Hum Mol Genet.* **16**, 942–56 (2007).

20. Hiesberger, T. *et al.* Proteolytic cleavage and nuclear translocation of fibrocystin is regulated by intracellular Ca2+ and activation of protein kinase C. *J Biol Chem* **281**, 34357–64 (2006).

21. Cai, Y. *et al.* Identification and characterization of polycystin-2, the pkd2 gene product. *J Biol Chem* **274**, 28557–28565 (1999).

22. Gainullin, V. G., Hopp, K., Ward, C. J., Hommerding, C. J. & Harris, P. C. Polycystin-1 maturation requires polycystin-2 in a dose-dependent manner. *The J. clinical investigation* **125**, 607–620 (2015).

23. Liang, G. *et al.* Polycystin-2 is regulated by endoplasmic reticulum-associated degradation. *Hum. molecular genetics* **17**, 1109–1119 (2008).

24. Roitbak, T., Surviladze, Z., Tikkanen, R. & Wandinger-Ness, A. A polycystin multiprotein complex constitutes a cholesterol-containing signalling microdomain in human kidney epithelia. *Biochem. J* **392**, 29–38 (2005). DOI 10.1042/BJ20050645.

25. Yoder, B. K., Hou, X. & Guay-Woodford, L. M. The polycystic kidney disease proteins, polycystin-1, polycystin-2, polaris, and cystin, are co-localized in renal cilia. *J Am Soc Nephrol* **13**, 2508–16 (2002).

26. Parnell, S. C. *et al.* The polycystic kidney disease-1 protein, polycystin-1, binds and activates heterotrimeric G-proteins in vitro. *Biochem. Biophys Res Commun* **251**, 625–31 (1998).

27. Parnell, S. C. *et al.* Polycystin-1 activation of c-Jun N-terminal kinase and AP-1 is mediated by heterotrimeric G proteins. *J Biol Chem* **277**, 19566–72 (2002).

28. Hackmann, K. *et al.* A splice form of polycystin-2, lacking exon 7, does not interact with polycystin-1. *Hum. molecular genetics* **14**, 3249–3262 (2005).

29. Q, S. *et al.* Structure of the human pkd1-pkd2 complex. *Sci. 2018 Sep 7;361(6406)* (2018).

30. Outeda, P. *et al.* A novel model of autosomal recessive polycystic kidney questions the role of the fibrocystin c-terminus in disease mechanism. *Kidney international* **92**, 1130–1144 (2017).

31. Hogan, M. C. *et al.* Pkhdl1, a homolog of the autosomal recessive polycystic kidney disease gene, encodes a receptor with inducible t lymphocyte expression. *Hum Mol Genet.* **12**, 685–698 (2003). (eng).

32. Huang, Y.-S., Chiang, N.-Y., Chang, G.-W. & Lin, H.-H. Membrane-association of emr2/adgre2-ntf is regulated by site-specific n-glycosylation. *Sci. reports* **8**, 4532 (2018).

33. Sapay, N., Guermeur, Y. & Deléage, G. Prediction of amphipathic in-plane membrane anchors in monotopic proteins using a svm classifier. *BMC bioinformatics* **7**, 255 (2006).

34. Wubbolts, R. *et al.* Proteomic and biochemical analyses of human B cell-derived exosomes. Potential implications for their function and multivesicular body formation. *J Biol Chem* **278**, 10963–72 (2003).

35. Ong, A. C., Harris, P. C., Biddolph, S., Bowker, C. & Ward, C. J. Characterisation and expression of the PKD-1 protein, polycystin, in renal and extrarenal tissues. *Kidney Int* **55**, 2091–2116 (1999).

36. Ward, C. J. *et al.* Polycystin, the polycystic kidney disease 1 protein, is expressed by epithelial cells in fetal, adult, and polycystic kidney. *Proc Natl Acad Sci U S A* **93**, 1524–8 (1996).

37. Cai, Y. *et al.* Calcium dependence of polycystin-2 channel activity is modulated by phosphorylation at ser812. *J. Biol. Chem.* **279**, 19987–19995 (2004).

38. Hogan, M. C. *et al.* Characterization of pkd protein-positive exosome-like vesicles. *J Am Soc Nephrol* (2009). DOI 10.1681/ASN.2008060564.

39. Chen, C. Y., Hogan, M. C. & Ward, C. J. Purification of exosome-like vesicles from urine. *Methods Enzym.* **524**, 225–241 (2013). DOI 10.1016/B978-0-12-397945-2.00013-5.

40. Wittig, I., Braun, H.-P. & Schägger, H. Blue native page. *Nat. protocols* **1**, 418 (2006).

Acknowledgments

This study was supported by the PKD Biomarkers and Biomaterials Core in the Kansas PKD Research and Translational Core Center (National Institutes of Health (NIH): National Institute of Diabetes and Digestive and Kidney Diseases [NIDDK] grant P30 DK106912).
Additional support was from National Institutes of Health NIDDK grants R01DK080688 ("Functional analysis of PKD proteins in urinary exosomes") and RC1DK086161 ("Analysis of the proteome of PKD–ELVs in polycystic kidney disease and controls").
E8 monoclonal antibody was provided by the NIDDK sponsored Baltimore Polycystic Kidney Disease Research and Clinical Core Center, P30DK090868". The address of the Core is 'University of Maryland School of Medicine Division of Nephrology, 655 W. Baltimore St. Baltimore, MD 21201'

Data availability statement

The entire data set is available at the ProteomeXchange.
ftp://ftp.pride.ebi.ac.uk/pride/data/archive/2014/12/PXD001075 accession PXD001075.

Author contributions statement

W.A.L., M.C.H., M.S., C.J.W. developed the intellectual basis for the paper, collected patients and developed the assays used. K.M., S.C.P. and L.Z. performed experiments. M.C.C., B.J.M., K.L.J. and D.J.M. performed the proteomics and the analysis of the data. All authors reviewed the manuscript.

Additional information

Competing Interests The authors declare no competing financial or non financial interests.

Human material

Urine was de-identified through the KUMC bio-specimens core, PI Prof. Darren Wallace. This core complies with federal regulations and was approved by the institutional review board at KUMC. This core (PKD Repository: HSC #13674; approval on July 9, 2014) closely adheres to Section 164.514(b)2 of the HIPAA Privacy Rule, which provides the standard for de-identification of protected health information (Safe Harbor method). Informed consent was confirmed for each individual that donated urine in accordance with the Declaration of Helsinki.

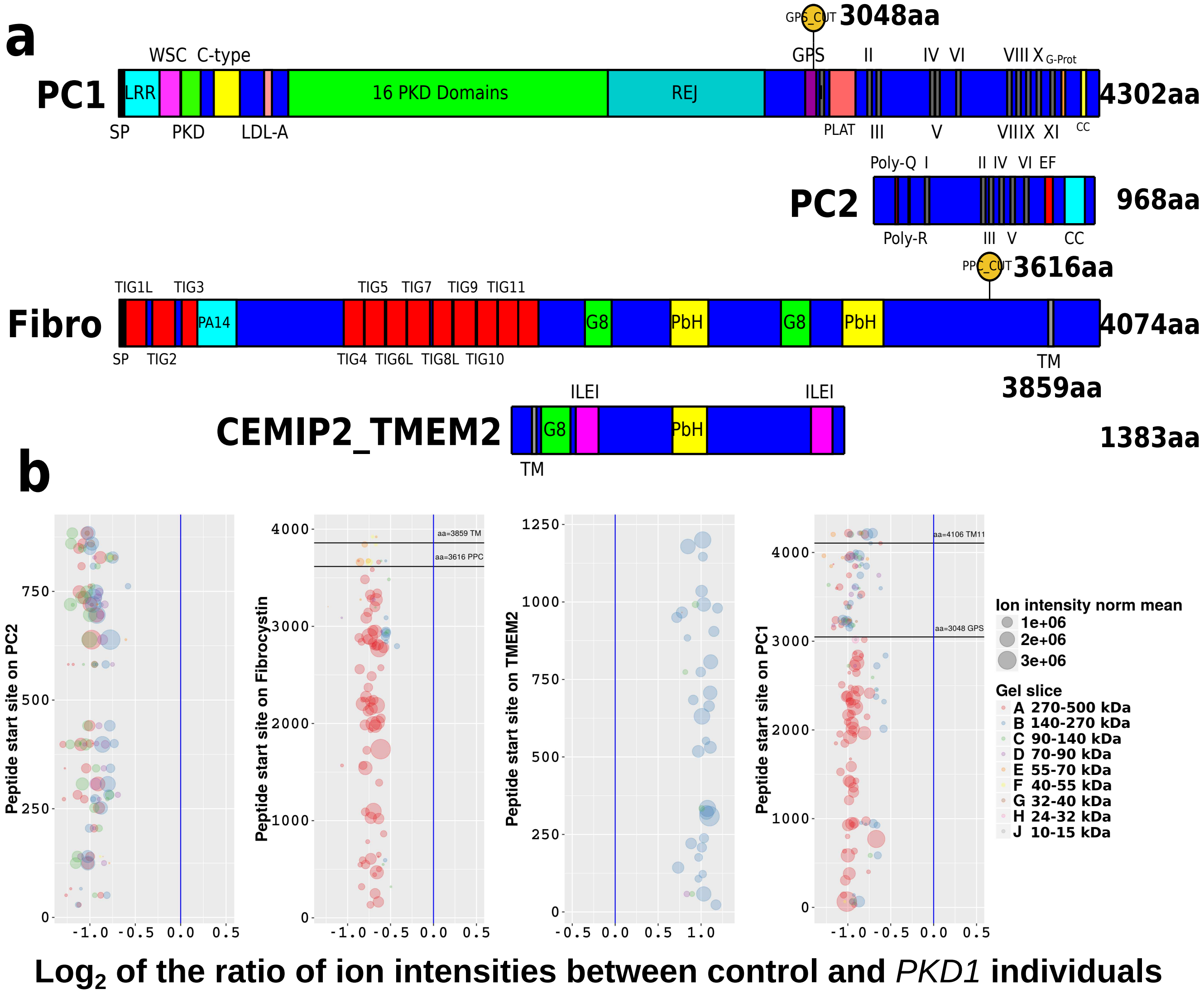

Figure 1. **Analysis of the polycystins, fibrocystin and CEMIPS_TMEM2:** a) Structures of the proteins investigated, polycystin-1 (PC1), polycystin-2 (PC2), fibrocystin (Fibro) and cell surface hyaluronidase (CEMIPS_TMEM2). b) Using peptide data from Elucidator (3.3.0.1.SP3.19) and re-analysis of Hogan *et al* 2014 data[5], we focused on peptides that definitely came from the PCC and we selected peptides that were decreased or increased with an uncorrected t–test p–value < 0.01. For proteins PC1, PC2, fibrocystin and CEMIPS_TMEM2, peptide starting positions were plotted on the y axis versus the $\log_2$ of the intensity ratio *PKD1*/normal, with point area scaling with intensity (ion current) and color indicating the gel slice. Gel slice A, 270 kDa–500 kDa; B, 140 kDa–270 kDa; C, 90 kDa–140 kDa; D, 70 kDa–90 kDa; E, 55 kDa–70 kDa; F, 40 kDa–55 kDa; G, 32 kDa–40 kDa; H, 24 kDa–32 kDa; I, 15 kDa–24 kDa; and J, 10 kDa–15 kDa[5]. Data in Suppl_Database_1.csv

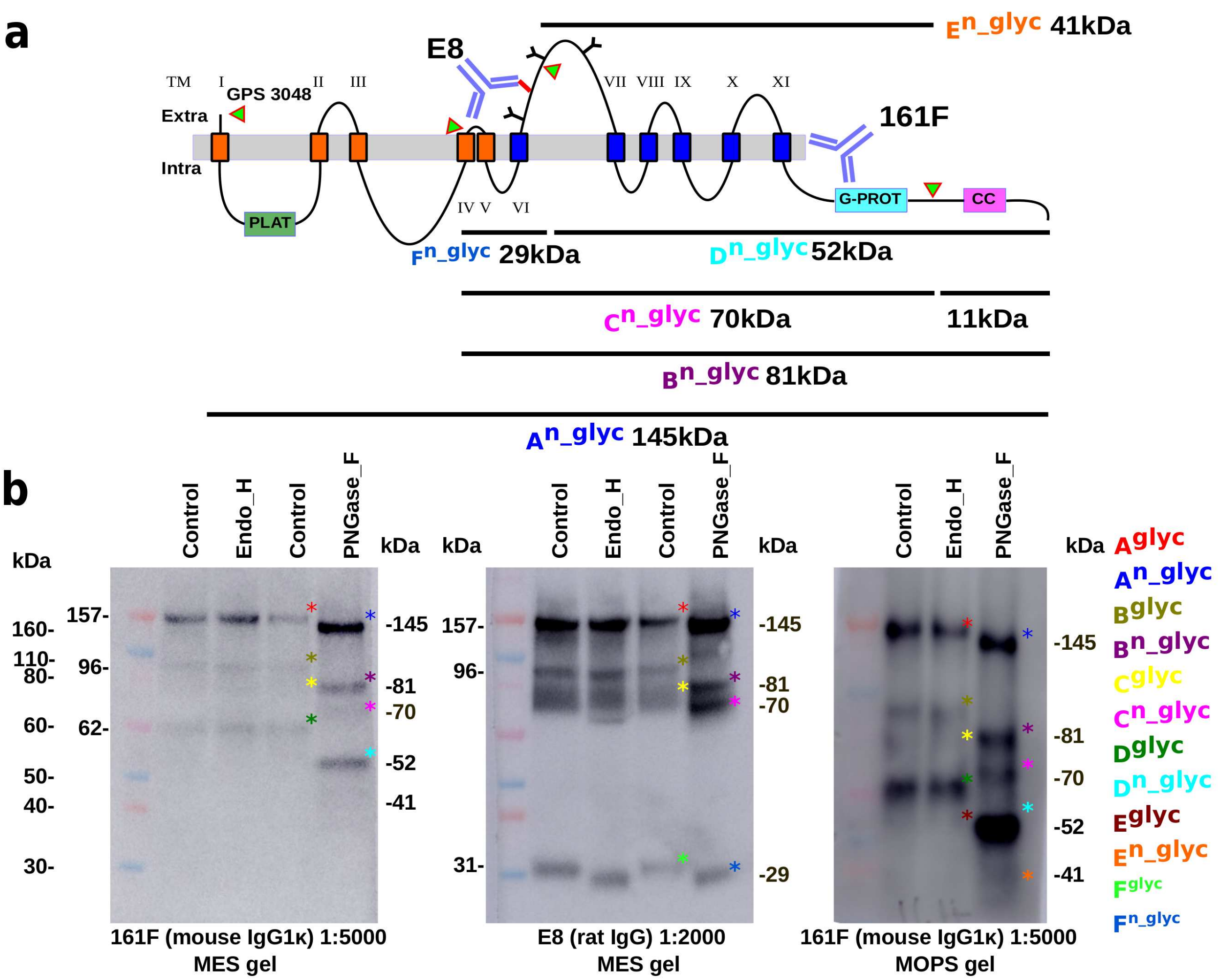

Figure 2. Analysis of the C–terminus of PC1 in ELVs: a) Schematic of the C–terminus of PC1 with cleavage events marked. b) Highly purified urine ELVs (fraction B from a D_2O 5–30% sucrose gradient) were run on 4–12% MES and MOPS gels. MES gels resolve lower molecular weight molecules well whereas MOPS has the opposite effect. Monoclonal antibody PKS–A (161F, IgG1κ) identifies an epitope between amino acids (4070..4156), between TM11 and coiled-coil domain in the C-terminal tail of PC1[36]. Rat monoclonal antibody E8 identifies a peptide epitope in the TM–VI to TM–VII extracellular loop. ELVs were treated with Endo H to remove ER type high mannose saccharides, or PNGase F to remove all N–linked saccharides. The various bands A–F observed on these western blots are color coded, ^{glyc} refers to the glycosylated form and $^{n\text{-}glyc}$ the deglycosylated form. Original westerns are in Supplemental_Data_Fig2.zip

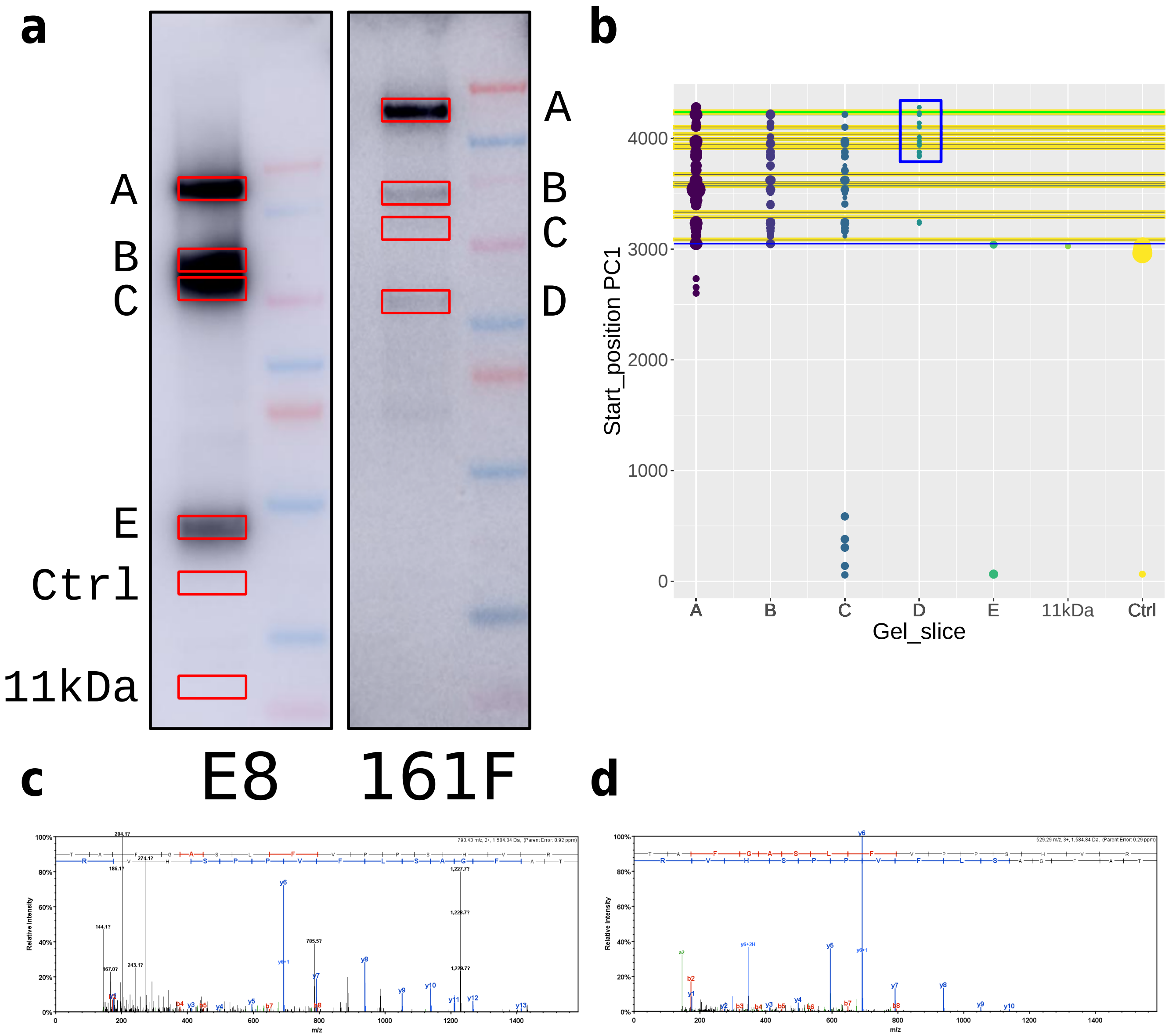

Figure 3. Analysis of the C–terminus of PC1 by MS/MS: a) Western blots on PNGase F treated ELVs probed with E8 (MES gel) and 161F (MOPS gel). A^{ms}–E^{ms} are the gel slices taken for proteomic analysis. Ctrl is a control gel slice and 11 kDa a gel slice taken to probe for extreme C–terminal peptide products of the C-terminal tail cleavage event. b) Distribution of peptides on the ORF of PC1 with point diameter scaling with ion intensity a rough proxy for abundance. Blue horizontal line at 3048 is the GPS cleavage site, the 11 yellow lines represent the TM domains and the final green line the coiled coil domain. Note the lack of peptides before aa 3048 (GPS cleavage). c–d) We were able to detect the peptide representing the GPS cleavage to the next C–terminal tryptic site **TAFGASLFVPPSHVR**, the N–terminal **T** is at 3049 in the **HL↑T** autocleavage site. There is a doubly charge ion at m/z=793.43 (c) and a triply charged ion at m/z=529.29 (d). Data in Suppl_Database_1A.csv

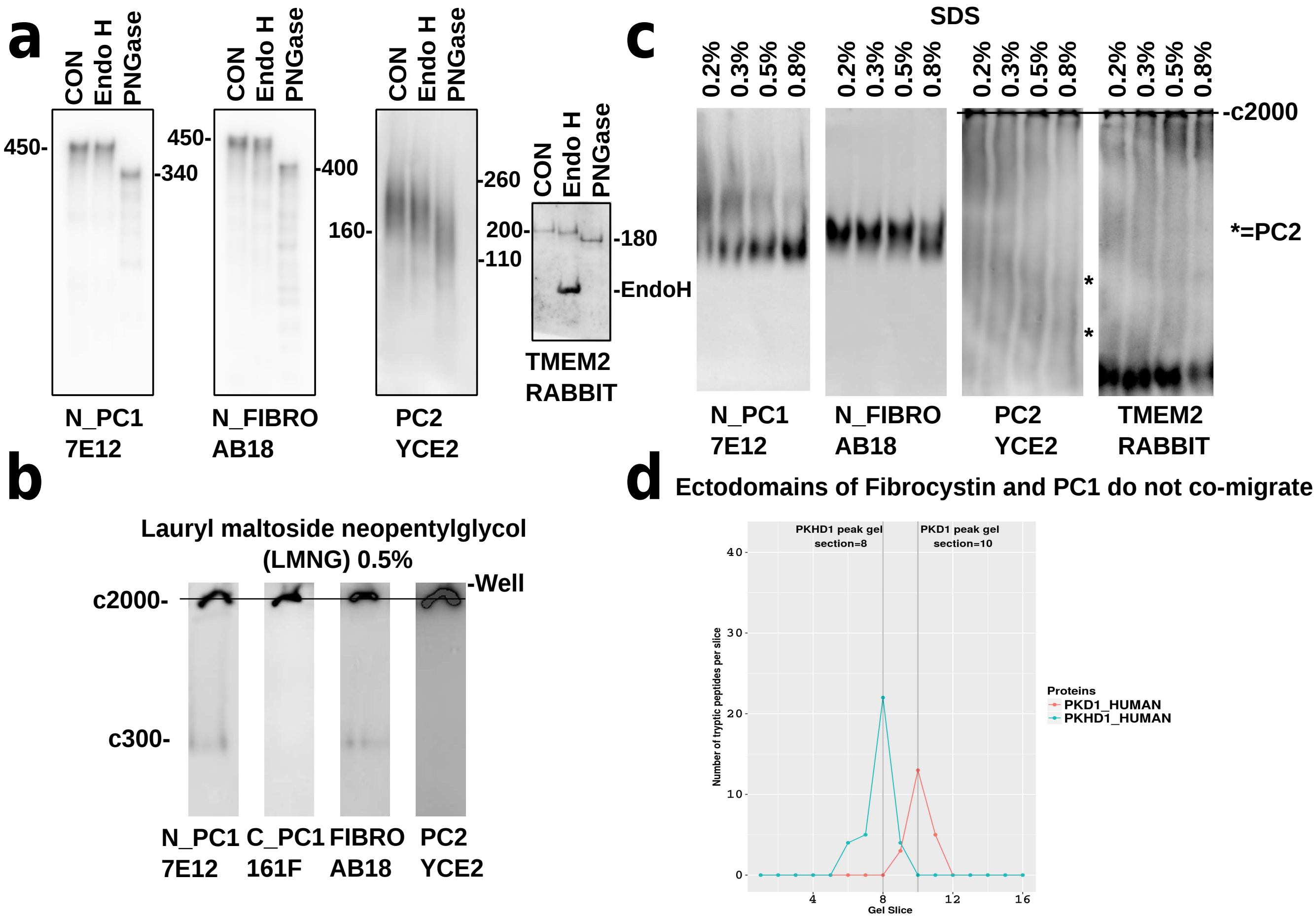

Figure 4. Western and MS/MS analysis of the PCC in ELVs: a) Conventional 3–8% SDS PAGE western blots of highly purified ELVs , probed with monoclonal antibodies again the N–terminus of PC1, N–terminus of fibrocystin and PC2 plus a polyclonal rabbit anti–CEMIPS_TMEM2. All proteins are sensitive to PNGase F but resistant to Endo H showing that they have Golgi type carbohydrate modifications and are by definition mature. b) Native gels performed with a mild detergent, lauryl maltoside neopentylglycol and a detergent that can interact with cholesterol, taurocholic acid. Amphipol A8–35 was used as a charge conferring molecule. These data showed that PC2 and the C–terminus of PC1 migrate only a short way into the gel about 5 mm (c2 MDa delineated by the black line) and a small amount of N–terminal PC1 and fibrocystin ectodomain migrate at c300 kDa. The same phenomenon is seen in 'blue native' electrophoresis (but has high background due to the Coomassie dye interacting with PVDF). c) In the presence of varying amounts of SDS (non reducing incubated on ice 20 min), 0.2%–0.8% the bulk of N–terminal PC1 and fibrocystin were freed from the complex in the well and migrates at c300 kDa. At low SDS levels the PC1 has a complex migration pattern implying that it exists in multiple complexes. PC2 runs in the high molecular 2 MDa complex and as a faint dimer and monomer asterisk. Most CEMIPS_TMEM2 resolves at c200 kDa. d) MS/MS analysis of 16 gel slices taken across the region containing the N–terminal PC1 and fibrocystin ectodomains shows that they do not resolve in the same slice and likely do not interact under these conditions. The PC1 ectodomain is smaller than fibrocystin's ectodomain. Original westerns are in Supplemental_Data_Fig3.zip and MS/MS data in Suppl_Database_2.csv
