# Supplementary material for: Analysis of the polycystin complex (PCC) in human urinary exosome–like vesicles (ELVs)
Source: Sci Rep. 2020 Jan 30;10:1500. doi: 10.1038/s41598-020-58087-3 (PMC6992733; doi:10.1038/s41598-020-58087-3)
Supplement: Supplementary file 2 — Supplementary Dataset 1. [file 41598_2020_58087_MOESM2_ESM.pdf]

Analysis of the polycystin complex (PCC) in human urinary exosome–like vesicles (ELVs).

January 2019

[1,+]Wendy A. Lea [1,+]Kerri McGreal [1+]Madhulika Sharma [1,2]Stephen C. Parnell [1]Lesya Zelenchuk [3]M. Cristine Charlesworth [3]Benjamin J. Madden [3]Kenneth L. Johnson [3]Daniel J. McCormick [4]Marie C. Hogan [1,*]Christopher J. Ward

[1]The Jared Grantham Kidney Institute, University of Kansas Medical Center, Kansas City, KS 66160, USA. [2]Department of Biochemistry and Molecular Biology, University of Kansas Medical Center, Kansas City, KS 66160, USA. [3]Mayo Proteomic Core, Medical Sciences Building, Ms 3-121, Mayo Clinic, 200 First Street, SW Rochester, MN 55905, USA. [4]Division of Nephrology, Department of Internal Medicine, Mayo Clinic, Rochester, USA. [*]cward6@kumc.edu

[+]These authors contributed equally to this work

1 Supplemental Data:

Suppl_fig_1

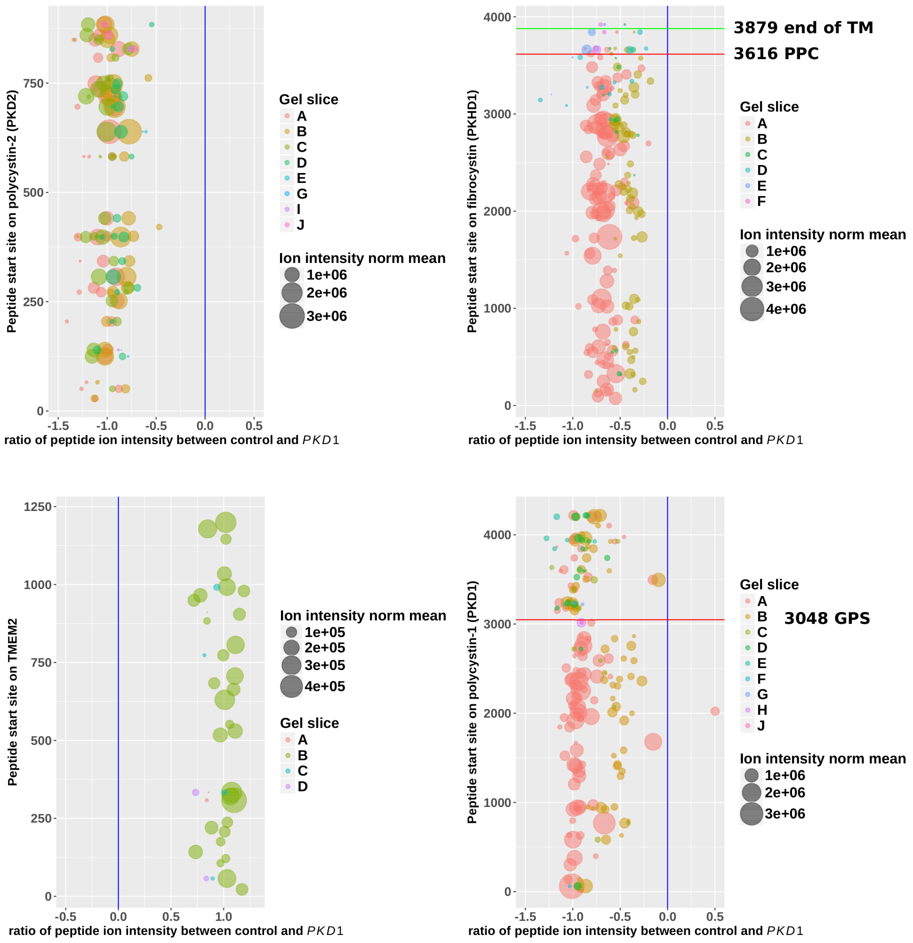

Figure 1: **Analysis of the polycystins, fibrocystin and CEMIPS_TMEM2:** Peptide start site vs $\log_2$ ratio ion intensity between control and individuals with PKD1 mutations. Size of bubble scales with the intensity of the peptide ion. Color coded for gel slice. In this case all peptides were used and there was no trimming for p-value (Normal vs PKD1). This is the same data as in figure 1 except there is no trimming for p-value

Suppl_fig_2

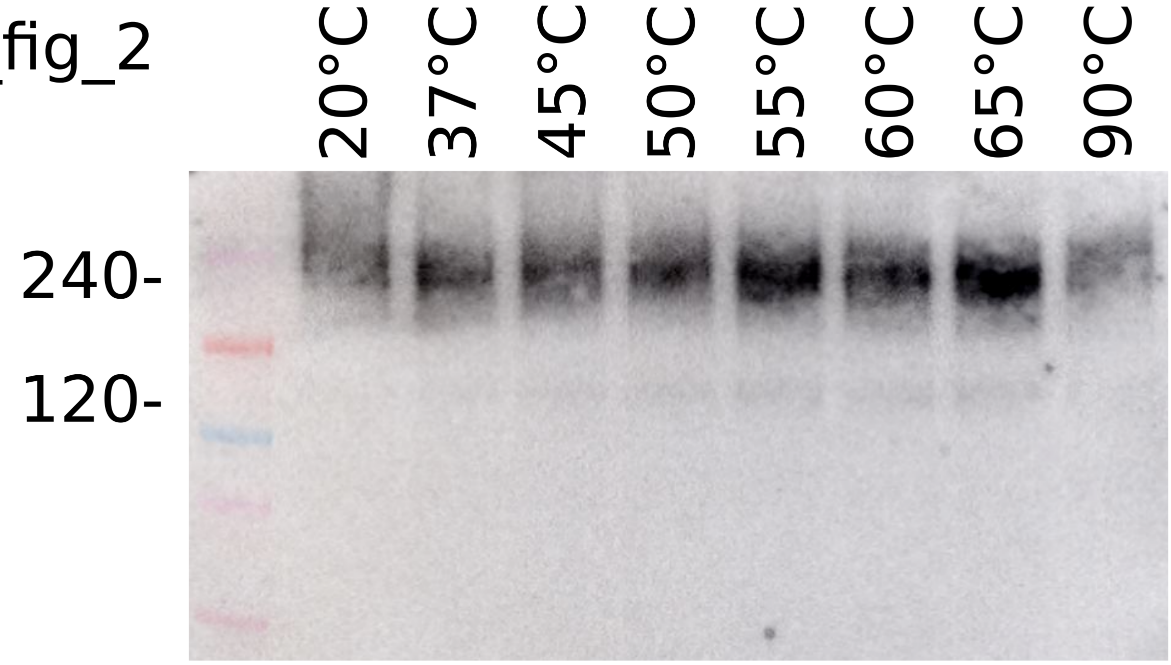

Exosomes incubated for 10 minutes in the presence of LiDS TCEP at various temperatures. Dimer resolves at 240kDa and monomer 120kDa. Antibody YCE2 1:1000 anti IgG2a-HRP secondary 1:2000.

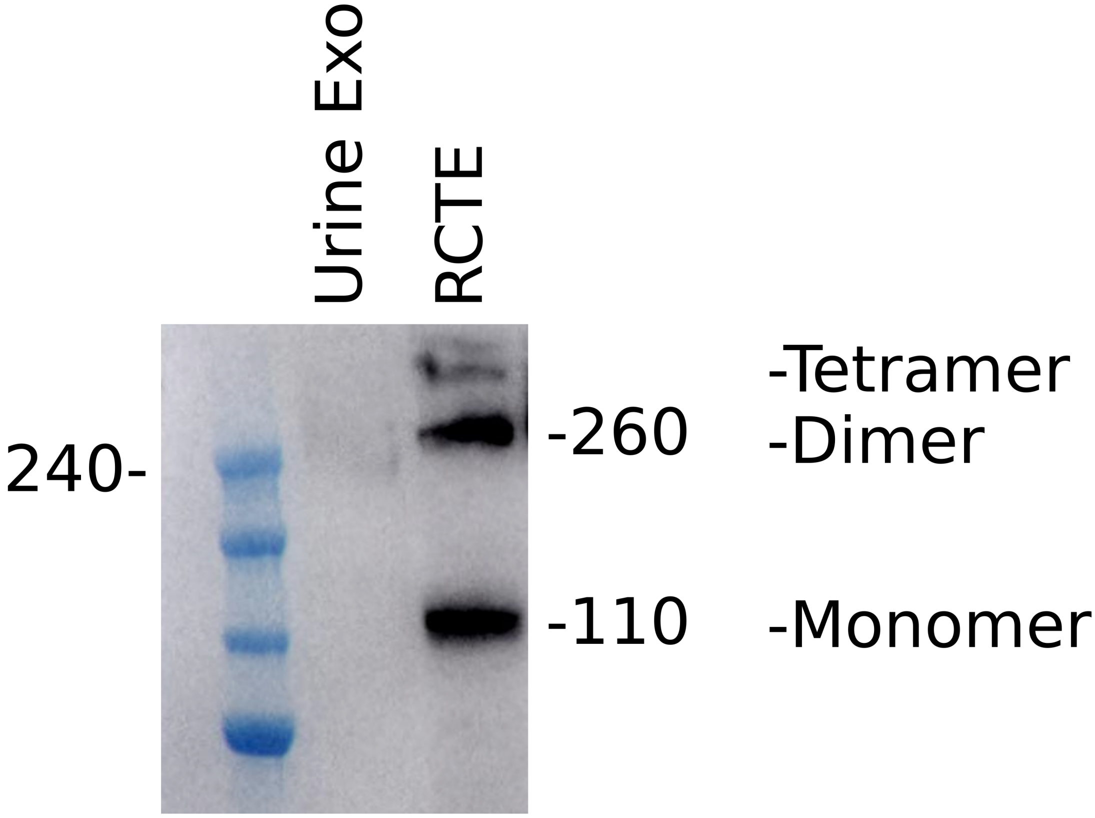

Human renal cortical collecting duct (RCTE) cells compared to human exosomes. Ab YCE2 as above. Temp 65°C LIDS TCEP.

Suppl_fig_3

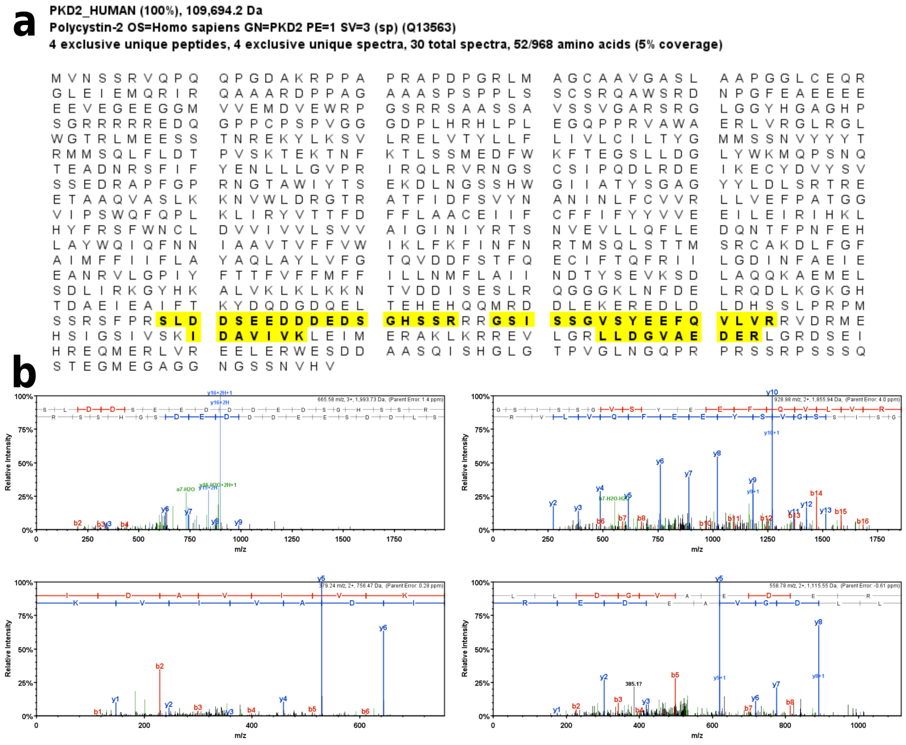

Figure 2: **The presence of C–terminal peptides from PC2 in gel sections I–J implies cleavage N–terminal to the coiled coil domain:** We detected 4 PC2 peptides from the extreme C–terminus of the protein in gel sections I and J <24kDa. This implies that about 13% of the protein underwent a cleavage event just N–terminal to the coiled coil domain of the protein.
